# Adverse impact of univentricular pacing for the patient with functional single ventricle: successful conversion to cardiac resynchronization therapy

**DOI:** 10.1186/s40792-020-00863-4

**Published:** 2020-05-13

**Authors:** Ai Sugimoto, Kiyohiro Takigiku, Shuichi Shiraishi, Masashi Takahashi, Masanori Tsuchida

**Affiliations:** 1 Division of Thoracic and Cardiovascular Surgery, Niigata University Graduate School of Medical and Dental Sciences, 1-757, Asahimachi-dori, Niigata City, 951-8510 Japan; 2 Department of Pediatric Cardiology, Nagano Children’s Hospital, Azumino City, Japan

**Keywords:** Cardiac resynchronization therapy, Pediatric, Congenitally corrected transposition of the great artery, Fontan procedure

## Abstract

**Background:**

In a Fontan candidate, univentricular pacing may cause delay in interventricular conduction, which induces asynchronous contraction. Cardiac resynchronization therapy is expected to be an effective mode of therapy in such a case.

**Case presentation:**

A 7-month-old girl, diagnosed with dextrocardia, congenitally corrected transposition of the great artery [situs solitus, L-loop, and L-transposition], ventricular septal defect, infundibular and pulmonary valvular stenosis, and straddling of the tricuspid valve, was considered as a candidate for the Fontan procedure. She had undergone Blalock-Taussig shunt, and epicardial univentricular pacemaker implantation for persistent complete atrioventricular block. She underwent a bidirectional cavopulmonary shunt concomitant with ventricular lead translocation from the morphological left ventricle to the morphological right ventricle. After discharge, ventricular dyssynchrony was noted and cardiac failure persisted. She was converted to cardiac resynchronization therapy (CRT) at 13 months of age. Two-dimensional speckle tracking imaging was used by cardiologists to determine the most suitable pacing site. CRT rapidly corrected the heart failure; thus, she underwent the Fontan procedure after 1.5 years. Five years have passed since the cardiac resynchronization therapy; her interventricular synchrony is maintained well and the level of brain natriuretic peptide remains within normal range.

**Conclusion:**

We describe the successful conversion from single ventricular pacing to CRT, in a case of congenitally corrected transposition of the great artery indicated for the Fontan procedure. The long-term prognosis of cardiac resynchronization therapy is undetermined in the pediatric population; therefore, further follow-up is required.

## Background

In patients with functional single ventricle, determination of the optimal pacing site is sometimes difficult. The negative impact of univentricular pacing on systemic ventricular function in congenitally corrected transposition of the great artery (ccTGA) has been reported [1]. In a Fontan candidate with two ventricles, univentricular pacing may cause delay in interventricular conduction, which induces asynchronous contraction. Cardiac resynchronization therapy (CRT) is expected to be an effective mode of therapy in such a case [2].

## Case presentation

**Fig. 1:**
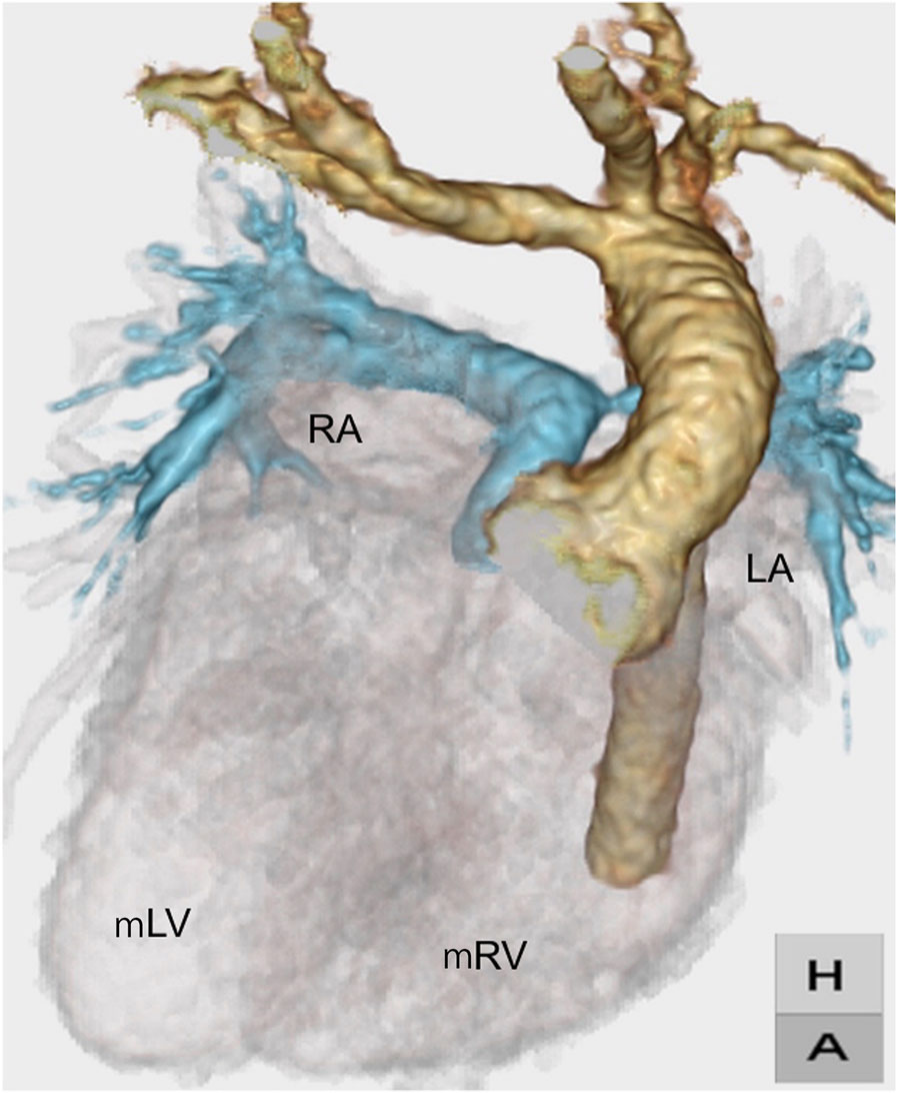
Three-dimensional computed tomography before initial palliation. LA, left atrium; mRV, morphological right ventricle; mLV, morphological left ventricle

A 7-month-old girl, weighing 5.4 kg, diagnosed with dextrocardia, ccTGA [situs solitus, L-loop, and L-transposition], ventricular septal defect, infundibular and pulmonary valvular stenosis, balanced ventricles (Fig. 1), was considered as a candidate for the Fontan procedure due to straddling of the tricuspid valve. She had undergone modified Blalock-Taussig shunt (BTS) for initial palliation at 4 months of age. Because of persistent complete atrioventricular block, which appeared at the induction of the anesthesia for BTS, she had undergone an epicardial univentricular pacemaker implantation on the morphological left ventricle (mLV), at 5 months of age. She presented with cardiac failure due to pulmonary over-circulation after BTS. Brain natriuretic peptide (BNP) at admission was 2552 pg/dl, and she could not leave tube feeding. She underwent a bidirectional cavopulmonary shunt with pulmonary stump closure. Ventricular septal defect was large enough. Ventricular lead was translocated from the mLV to the morphological right ventricle (mRV) by a request from pediatric cardiologists. She was discharged once. Her BNP decreased to 212 pg/dl, and she left tube feeding 1-month postoperatively. However, her BNP started to rise again; it reached as high as 1128 pg/dl, regardless of pharmacotherapy. And remarkable ventricular dyssynchrony was noted.

**Fig. 2:**
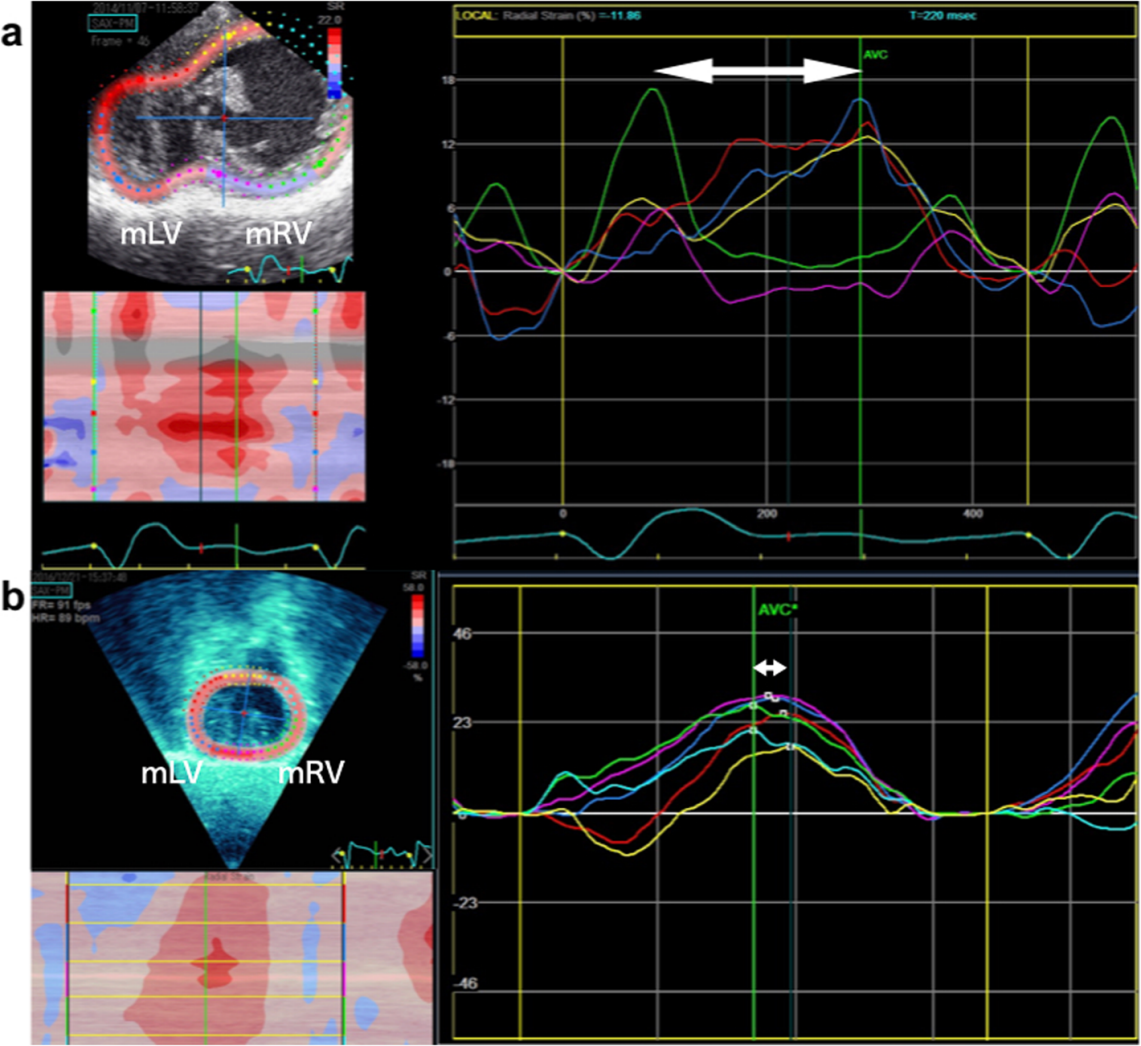
**a** Speckle tracking echocardiography reveals that the difference in time to peak radial strain between both ventricles is 210 ms before CRT (white arrow). **b** Speckle tracking echocardiography after CRT shows good interventricular synchrony between both ventricles. The peak radial strain between both ventricles is very short (white arrow). CRT, cardiac resynchronization therapy; mLV, morphological left ventricle; mRV, morphological right ventricle

**Fig. 3:**
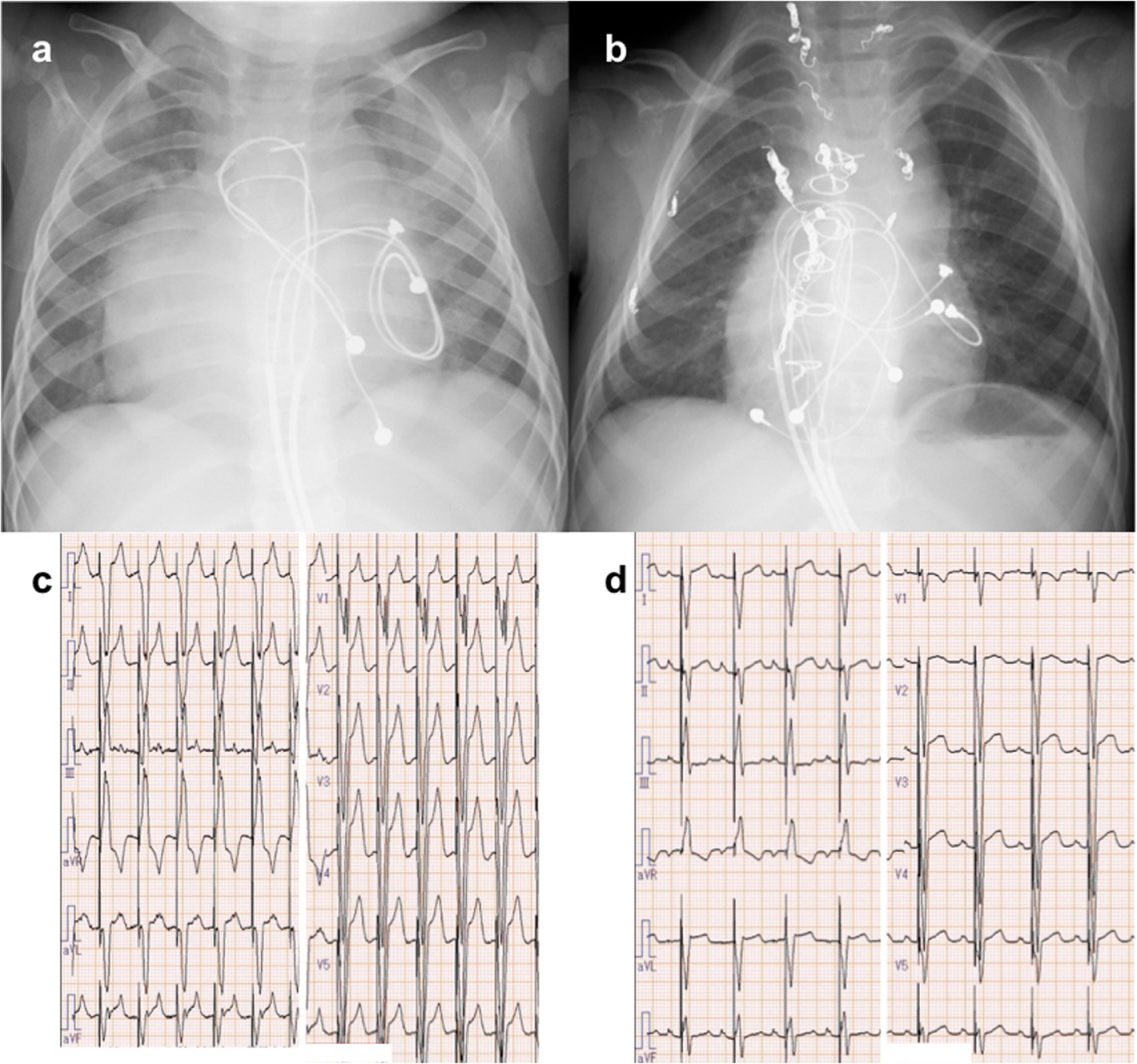
**a** Chest radiograph before cardiac resynchronization therapy (CRT). The cardiothoracic ratio is 65%. Atrial leads are placed on the LA and ventricular leads on the mRV. **b** Chest radiograph post-CRT. The cardiothoracic ratio is reduced to 50%. New ventricular leads are placed on the mLV, and the cathode of the previous mRV pacing lead is re-located to the mRV outflow tract. **c** Electrocardiogram before CRT. QRS duration is 156 ms. **d** Electrocardiogram after CRT. QRS duration is shortened to 140 ms. CRT, cardiac resynchronization therapy; LA, left atrium; mRV, morphological right ventricle; mLV, morphological left ventricle

CRT was planned at 13 months of age. After the chest was reopened and the heart was dissected, epicardial echocardiography was performed by pediatric cardiologists using two-dimensional speckle tracking imaging. Figure 2 a shows speckle-tracking radial strain delay before CRT, which was 210 ms between both ventricles. To determine the most effective pacing site, initially, we searched the best pacing site that improved the mLV contraction on the mLV lateral side. Thereafter, we proceeded to adjust the mRV pacing site. We searched the pacing site on the opposite side of the mLV pacing site, where the ventricular synchrony would be improved the most. An accurate adjustment of the position in the mRV pacing site induced a dramatic change in the ventricular synchrony. A CRT-pacemaker (Viva CRT-P C5TR01, Medtronic®, MN) was implanted. The initial CRT setting was as follows: DDD, lower rate 120 bpm; A-V delay, 120 ms; and V-V delay, 0 ms. The following medications were administered: pimobendane 0.1 mg/kg/day, nicorandil 0.5 mg/kg/day, and digoxin 0.01 mg/kg/day. The dose of carvedilol, which was already administered prior to CRT, was gradually increased to 0.1 mg/kg/day. The dose of digoxin was adjusted under therapeutic drug monitoring; blood concentration of digoxin was 0.5 ng/ml at discharge. The setting of CRT was adjusted to the patient using transthoracic speckle tracking echocardiography every week after the patient left the intensive care unit. She was discharged on the 29th postoperative day. The final CRT setting at discharge was as follows: DDD, lower rate 110 bpm; A-V delay, 120 ms, mRV preceding mLV; V-V delay, 20 ms. Ventricular synchrony showed marked improvement postoperatively. BNP was decreased dramatically to 20 pg/dl at 1-month post-CRT. Figures 3 a to d show the pre and post-CRT chest radiographs and electrocardiogram (ECG). Cardiothoracic ratio improved from 65 to 50%, and QRS duration was reduced from 156 ms to 140 ms. The patient underwent the Fontan procedure 1.5 years after CRT insertion. Recent echocardiography (26 months after CRT) revealed good interventricular synchrony between both ventricles (Fig. 2 b). Five years following CRT, the patient’s interventricular synchrony is well maintained, and her BNP is within the normal limits. The consent for publication of this case and associate image was obtained from the parents at the outpatient clinic.

## Discussion

We reported a case of a ccTGA, in which univentricular pacing caused severe cardiac failure related to interventricular dyssynchrony. CRT played a significant role in the management of severe cardiac failure. The cardiac function of the patient has been well maintained more than 5 years post-CRT.

Negative impact of univentricular pacing on systemic ventricular function in ccTGA has already been reported. Hofferberth, et al. recommended primary biventricular pacing for all patients with ccTGA who develop heart block, as this prevents late systemic ventricular dysfunction [1]. Asynchronous contraction of the two ventricles is noted as a significant factor affecting cardiac output after the Fontan procedure in patients with biventricular heart [3]. Higaki et al. reported that the delay in interventricular conduction induced by univentricular pacing caused asynchronous contraction in a Fontan candidate with a biventricular heart. This causes the interventricular bidirectional flow due to communicating ventricular septal defect; it lowers the total cardiac output. They noted that CRT, which reduces the conduction delay between two ventricles, is expected to be an effective mode of therapy in such a case [2].

Identification of the optimal pacing site is important for optimal response to CRT [4]. In patients with CHD, variation of ventricular morphologies and the type of dyssynchrony makes determination of the optimal pacing sites difficult. Recently, the role of image-guided lead placement in CRT has been reported in the adult population [5]. However, in small children, speckle tracking imaging using trans-esophageal echocardiography is not available. In the previous reports in CRT for children, the way of determination of the pacing sites was described as using epicardial echocardiography [6] or intraoperative ventricular wall mapping using ECG [7]. In this study, intraoperative epicardial echocardiography using two-dimensional speckle tracking imaging enabled accurate determination of the correct pacing site, which leads to an excellent treatment outcome. Even if CRT simulation by catheter examination was performed before the CRT, epicardial echocardiography should be performed because it might be more accurate. Further adjustments of the setting of CRT are very important. In the present case report, the setting of CRT was adjusted every week; the setting of CRT at discharge was different from the beginning.

The best timing of the CRT might be right after the BDG, because cardiac dyssynchrony appeared after the BDG. It was better not to wait for cardiac failure to be worsened. It took us 6 months because we did not have pediatric CRT as a treatment option at that time. It is ideal to introduce CRT as a practical treatment option, because it is very effective method in some selected pediatric patients with severe heart failure.

## Conclusions

We described the successful conversion from single ventricular pacing to CRT, in a case of ccTGA indicated for the Fontan procedure, with severe cardiac failure owing to interventricular dyssynchrony which was caused by univentricular pacing. The long-term prognosis of CRT is undetermined in the pediatric population; therefore, further follow-up is required.

## Data Availability

Not applicable.

## Abbreviations

ccTGA: Congenitally corrected transposition of the great artery
CRT: Cardiac resynchronization therapy
BTS: Modified Blalock-Taussig shunt
mLV: Morphological left ventricle
BNP: Brain natriuretic peptide
mRV: Morphological right ventricle
ECG: Electrocardiogram
